# A cross-sectional study of secondhand tobacco exposures and attitudes towards Senate Bill 793 in Latino mothers and middle and high school students in the San Francisco Bay Area

**DOI:** 10.18332/tid/216183

**Published:** 2026-06-11

**Authors:** Kimberly Michel, Janet M. Wojcicki

**Affiliations:** 1 Department of Pediatrics, University of California, San Francisco, San Francisco, United States; 2 Department of Epidemiology and Biostatistics, University of California, San Francisco, United States

**Keywords:** e-cigarettes, ENDS, vapes, flavored tobacco, Latino youth

## Abstract

**INTRODUCTION:**

Secondhand smoke (SHS) and aerosol (SHA) exposures continue to be at unacceptably high levels in California, including among Latino communities, increasing the risk for chronic disease. California enacted legislation to reduce tobacco use and initiation, particularly among adolescents, through Senate Bill (SB) 793 which forbids the sale of flavored tobacco products in California. Our study evaluated exposures to smoking, including e-cigarette use and combustible smoking, and SHS and SHA among primarily foreign-born, Spanish-speaking Latina mothers and US-born middle and high school children via a survey in the greater San Francisco Bay Area in 2023–2024.

**METHODS:**

Using a longitudinal cohort of Latina mothers and their middle and high school children, surveys were used to assess attitudes towards SHS and SHA exposures and SB 793 including the ability to purchase flavored tobacco products. Means and standard deviations were used to characterize continuous predictors and outcomes and percentages for categorical ones. T-tests, Fisher's exact and chi-squared tests were use to assess possible associations between demographics and outcomes. All data was analyzed cross-sectionally.

**RESULTS:**

In a sample of 115 predominantly Spanish-speaking mothers and US-born high school (n=50) and middle school (n=30) students in the greater San Francisco Bay Area, we found that >50% of high school students reported SHS and/or SHA exposures in the last 7 days. Unlike their mothers and middle school students, high school students often had exposures at school (in bathrooms, walkways and outdoors). High school students avoided SHS and SHA less than their mothers (34% vs 12%; p<0.01). High school students were more skeptical that SB 793 had changed the ability to source flavored tobacco products than their mothers or middle school students who believed in the efficacy of the law.

**CONCLUSIONS:**

Future interventions should target students prior to high school as there were significant differences in attitudes based on age group. High schools have an unacceptably high amount of SHS and SHA exposure, and future interventions need to be high school-based.

## INTRODUCTION

### Secondhand smoke and aerosol in California

In spite of on-going attempts to limit outdoor exposures to secondhand smoke (SHS) and aerosol (SHA) in California through statewide and municipal ordinances, the California Youth Tobacco Survey 2024 found that >60% of high school students were exposed to SHS or SHA in the past two weeks in an outdoor location, with a higher percentage of Latino adolescents exposed compared with Asians, African-Americans and those that identified as Other^1^. Exposure to SHS and SHA may be particularly concerning for Latinos in California as 44% live in areas with poor air quality compared with 25% of other ethnic and racial groups^2^. Additionally, Latino children under 10 years of age make up 81% of those living in the most polluted areas.

### Senate Bill (SB) 793

In efforts to reduce the burden of chronic disease related to tobacco use, California has made attempts to decrease the overall tobacco product use and initiation, particularly among adolescents. California voters passed Senate Bill (SB) 793 in 2022, which prohibits the sale of flavored tobacco products in California. This law went into effect on 1 January 2023^3^. In 2024, electronic nicotine delivery systems (ENDS), including e-cigarette product use, remained the most commonly used tobacco product type for US adolescents^4^.

SB 793 has the potential to dramatically reduce ENDS product use among Californian adolescents. Previous studies have suggested that youth prefer flavored products with 79.1% of US adolescent users reporting flavored consumption^5^. Meanwhile, a study conducted pre- and post-flavored tobacco ban in California found that while SB 793 was effective in reducing some types of flavored tobacco use (combustible cigarettes, cigars and smokeless tobacco), flavored e-cigarette use in adolescents was unchanged stressing the need to continue to focus public health efforts in this population group^6^. Other studies similarly found that SB 793 had not impacted adolescent on-line sales of e-cigarettes in California^7^; and the 2024 California Youth Tobacco Survey found that high school students were continuing to purchase e-cigarettes from tobacco, smoke and vape shops (12th graders) compared with 10th grade students who sourced them most often from other individuals^1^.

### COVID-19

SB 793 was passed approximately 2 years after the COVID-19 pandemic ‘stay at home’ orders, which some studies suggested resulted in a decreased use of tobacco products early in the pandemic^8^, but returned to pre-pandemic levels by 2021 and 2022^9^. Any future interventions targeting SHS, SHA and tobacco product use, may benefit from thinking about exposures in the context of being in the post-COVID-19 era.

Using two established birth cohorts of primarily low socioeconomic status, foreign-born, Spanish-speaking Latina mothers and US-born Latino middle and high school adolescents in the greater San Francisco Bay Area, we sought to evaluate attitudes and practices of ENDS product and combustible cigarette use and SHS and SHA exposures in 2023 and 2024, after the implementation of SB 793. Second, we evaluated whether COVID-19 impacted any beliefs or attitudes towards use of tobacco products and SHS and SHA exposures as described below.

## METHODS

We conducted a cross-sectional survey of 115 Latina mothers, 50 high school children, and 30 middle school students on questions related to tobacco product use and SHS and SHA exposure using two previously recruited cohorts of San Francisco based-Latino mothers and children. Specifics of cohort recruitment and demographics collected have been previously described^10,11^. In short, pregnant Latina women were recruited at two San Francisco hospitals, University of California, San Francisco (UCSF) and Zuckerberg San Francisco General Hospital (ZSFG). We collected demographics on the participants at cohort recruitment including the following: language used at home (Spanish vs English, or English with Spanish); Special Supplemental Nutrition Program for Women, Infants and Children (WIC) participation during pregnancy (yes, no); maternal education level (high school or lower vs any college or higher), maternal age at the time of delivery (continuous and categorical with groupings as follows: 18–24, 25–30, or >30 years); residency in the US (<5, 5–15, >15 years), and child sex at birth (male, female). During the COVID-19 pandemic (2020 and 2021), families were re-contacted and questions were asked about number of individuals living in the household (continuous count and the following groupings: 1–3, 4–5 and ≥6) and maternal employment status (yes, no)^12^.

Participants were re-contacted by phone in 2023 and 2024, and mothers and children were invited to participate in this study which included asking questions from three validated surveys over the phone. Each interview took approximately 20 minutes and was conducted either in English or Spanish depending on the participant’s choice. The following surveys were used in the interview: 1) Adult Secondhand Exposure to Smoke (ASHES) questionnaire^13^, which focused on self-reported exposures to SHS and SHA (from any ENDS product) in different settings during two time periods – the last 24 hours and the last seven days – as well as an open-ended question that asked where any exposures had occurred; 2) Adolescent Smoking Consequences Questionnaire (ASCQ)^14^, which focused on consequences related to smoking cigarettes, with responses scaled from 1 (never) to 5 (always). All questionnaires have been previously validated with good measures of internal consistency. Additional questions were asked that assessed current tobacco use status, knowledge related to California Senate Bill 793^3^ and questions about any behavior changes with regard to SHS and SHA in relation to COVID-19. Child age at interview was also collected (in years) and current maternal and child tobacco product use.

To describe the sample, we calculated frequencies and percentages, and mean and standard deviation (SD) in relation to family and child demographics. Means and standard deviations were used to describe mother’s years in the US, the number of people in the home, child age, and mothers’ age at delivery.

Four groups were used as exposures for the study: high school students, middle school students, mothers of high school students, and mothers of middle school students. Several outcomes were measured, including self-reported tobacco use and attitudes to SHS and SHA, behavior changes related to changes in California legislation (SB 793), and beliefs regarding smoking, SHS and SHA, and the risk of COVID-19 and severe COVID-19. We did not analyze all portions of the ASHES or ASCQ, and as such no sub-scores or total scores were assessed. The study was approved by the Institutional Review Board (IRB) at the University of California, San Francisco (UCSF) and all mothers provided written consent for their and their children’s participation, and children gave assent to their participation.

### Statistical analysis

We used chi-squared and Student’s t-test and Fisher’s exact test to compare attitudes and beliefs between mothers and children in the high school versus middle school groups. All statistical tests were two-tailed, and comparisons between mothers and children were conducted separately for high school versus middle school samples. All analyses were conducted using R Studio version 2024.9.0.375 and Stata 18.0. Statistical significance was set for all analyses at p<0.05.

## RESULTS

### Sample demographics

A total of 115 mother and child pairs including 30 middle school and 50 high school students, respectively, were included in this study. The majority of mothers in both the high school group and the middle school groups primarily spoke Spanish at home (90.7% and 95.0%, respectively), with a small proportion speaking English (9.3% and 5.0%, respectively) (Table 1). High school student households tended to have a greater number of individuals living in the home than middle school families with more households with ≥6 individuals versus middle school households (30.8% vs 17.9%; p<0.01) (Table 1). Mothers of high school students were also older (29.3 ± 5.7 vs 25.9 ± 5.1 years, p=0.002) than those of middle school students at the birth of their child and more likely to be employed (50.0% vs 6.7%, p<0.01) (Table 1). Most mothers in the high school sample (89.39%) and almost all mothers in the middle school group (97.50%) utilized the WIC program during pregnancy (Table 1). A small percentage of both groups of mothers reported any smoking or use of tobacco products: 5.3% of high and 5.0% of middle school mothers. Similarly, a low percentage of children reported any smoking or use of tobacco products (2% of high and 0% of middle school students). The cohort of students was 42.7% and 56.7% female for middle and high school students, respectively, with the mean age of middle school students 11.11 ± 0.37 years versus 17.06 ± 0.27 years for high school students.

**Table 1:** Maternal and child demographics (N =115)

*Variables*	*High school group (N=75)*	*Middle school group (N=40)*	p*
	*n (%)*	*n (%)*	
***Family household variables***			
**Home language**			
English or Spanish/English	7 (9.3)	2 (5.0)	0.493
Spanish	68 (90.7)	38 (95.0)	
**People in the home**			**0.001**
Mean ± SD	4.9 ± 1.4	4.4 ± 2.3	0.17
1–3	9 (13.8)	19 (48.7)	**<0.001**
4–5	36 (55.4)	13 (33.3)	**0.027**
≥6	20 (30.8)	7 (17.9)	0.170
***Maternal variables***			
**Age at delivery** (enrollment) (years)			**0.008**
Mean ± SD	25.9 ± 5.1	29.3 ± 5.7	**0.002**
18–24	37 (49.3)	8 (20.5)	**0.049**
25–30	22 (29.3)	15 (38.7)	0.069
>30	16 (21.3)	16 (41.0)	**0.001**
**Current smoker**			
Yes	4 (5.3)	2 (5.0)	1.000
No	71 (94.7)	38 (95.0)	
**Education level**			
High school or lower	58 (78.4)	33 (84.6)	0.467
Any college or higher (graduate school or degree)	16 (21.6)	6 (15.4)	
**Employment**			
Yes	5 (6.7)	20 (50)	**<0.001**
No	70 (93.3)	20 (50)	
**WIC participation in pregnancy**			
Yes	59 (89.4)	39 (97.5)	0.254
No	7 (10.6)	1 (2.5)	
**Years in the US at birth of child**			
<5	15 (20)	6 (20)	1.000
5–15	44 (58.7)	19 (63.3)	0.826
>15	16 (21.3)	5 (16.7)	0.788
***Child variables***			
**Age at interview** (years), mean ± SD	11.11 ± 0.37	17.06 ± 0.27	<0.01
**Any self-reported tobacco use**			
Yes	1 (2)	0 (0)	1.000
No	49 (98)	30 (100)	
**Sex at birth**			
Male	43 (57.3)	13 (43.3)	0.204
Female	32 (42.7)	17 (56.7)	

WIC: Special Supplement Program in Nutrition for Women, Infants and Children.

* Statistical tests used included chi-squared, Student’s t-test and Fisher’s exact.

### Self-reported tobacco use and attitudes to secondhand smoke and aerosol exposures

**Figure 1:**
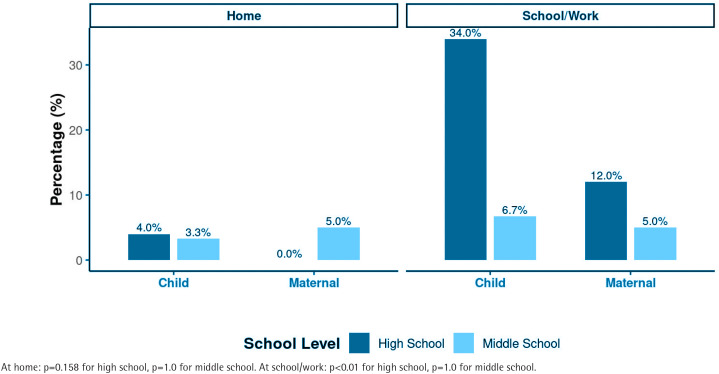
Percent of mothers and children that allow tobacco use around them

There were no statistically significant differences between high school children and parents in terms of having friends or family members who used tobacco products, while middle school students had fewer friends that were smokers compared with their mothers (0% vs 37.5% respectively) (Table 2). High school students (34%) were more likely to allow others to use tobacco products around them (including ENDS products) at school, when compared to the frequency their mothers (12%), allowed others to use tobacco products around them at work (p<0.01) (Table 2 and Figure 1).

**Table 2:** Maternal and child characteristics and attitudes and behavior related to smoking and secondhand smoke (SHS) and aerosol (SHA) exposures (N=115)

*Variables*	*High school group (N=75)*			*Middle school group (N=40)*		
	*Maternal n (%)*	*Child n (%)*	p*	*Maternal n (%)*	*Child n (%)*	p*
***Smoking demographics***						
**Total number of smokers in social and family circles**						
Family members (at least 1)	14 (18.7)	11 (14.7)	0.48	7 (17.5)	3 (10)	0.29
Friends (at least 1)	24 (32.0)	18 (24.0)	0.10	15 (37.5)	0 (0)	**<0.001**
**Rules about tobacco use in house/apartment building**						
No use anywhere	63 (87.5)	45 (90)	0.78	33 (82.5)	27 (90)	0.50
Some use is allowed	9 (12.5)	5 (10)		7 (17.5)	3 (10)	
***SHS and SHA exposures***						
**Self-reported exposures last 7 days to SHS or SHA**						
Home	4 (5.3)	5 (10)	0.482	5 (12.5)	2 (6.7)	0.691
Public place	32 (42)	26 (52)	0.39	21 (52.5)	13 (43.3)	0.478
Bus/car	8 (10.5)	10 (30)	0.7	2 (5)	0 (0)	
School		12 (24)			0 (0)	
Other	18 (24)	18 (36)	0.509	16 (40)	10 (33.3)	0.624
**Self-reported exposures last 24 hours to SHS or SHA**						
Home	3 (4)	1 (2)	0.649	4 (10)	1 (3.3)	0.383
Bus/car/public place	15 (197)	5 (10)	0.239	7 (23.0)	2 (7.1)	0.397
***Attitudes to SHS and SHA***						
**Allow others to use tobacco products around you at home**						
Yes/sometimes	0 (0)	2 (4)	0.158	2 (5)	1 (3.3)	1
No/never	75 (100)	48 (96)		38 (95)	29 (96.7)	
**Allow others to use tobacco products around you at school/work**						
Yes/sometimes	9 (12.0)	17 (34.0)	**<0.01**	2 (5)	2 (6.7)	1
No/never	66 (88.0)	33 (66.0)		38 (95.0)	28 (93.3)	
**Avoid smoke around you (move away)**						
Always	65 (86.7)	38 (76.0)	0.153	37 (92.5)	28 (93.3)	1
Sometimes/never	10 (13.3)	12 (24.0)		3 (7.5)	2 (6.7)	
**Avoid aerosol from ENDS around you (move away)**						
Always	66 (88.0)	37 (74.0)	**0.056**	37 (92.5)	29 (96.7)	0.63
Sometimes/never	9 (12.0)	13 (26.0)		3 (7.5)	1 (3.3)	
**Move away from smoke/aerosol around you**						
Always	66 (88.0)	35 (70.0)	**0.02**	37 (92.5)	28 (93.3)	1
Sometimes/never	9 (12.0)	15 (30.0)		3 (7.5)	2 (6.7)	
**Move away from smoke in the park**						
Always	69 (93.2)	45 (90.0)	0.754	37 (92.5)	28 (93.3)	1
Sometimes/never	6 (8.1)	5 (10.0)		3 (7.5)	2 (6.7)	
**Move away from aerosol in the park**						
Always	68 (90.7)	43 (86.0)	0.564	37 (92.5)	28 (93.3)	1
Sometimes/never	7 (9.3)	7 (14.0)		3 (7.5)	2 (6.7)	
***COVID and attitudes to smoking and vaping***						
**Believes smoking is more dangerous now because of COVID**						
Always	49 (68.1)	24 (48)	**0.038**	28 (70.0)	22 (73.3)	0.796
Sometimes/never	23 (31.9)	26 (52)		12 (30.0)	8 (26.7)	
**Believes smoking + ENDS increases risk of severe COVID**						
Always	41 (57.8)	17 (34)	**0.016**	23 (60.5)	19 (63.3)	0.244
Sometimes/never	30 (42.3)	33 (66)		15 (39.5)	11 (36.7)	
**Believes SHS can make it easier to get COVID**						
Always	28 (37.3)	12 (24)	0.170	16 (40)	11 (36.7)	0.809
Sometimes/never	47 (62.7)	38 (76)		24 (60)	19 (63.3)	
**Believes exposure to SHA can make it easier to get COVID**						
Always	27 (36)	12 (24)	0.173	14 (35)	12 (40)	0.8033
Sometimes/never	48 (64)	38 (76)		26 (65)	18 (60)	
***Attitudes towards SB 793***						
**Agrees with law prohibiting flavored tobacco sales**						
Always	63 (84)	40 (81.6)	0.808	32 (80)	26 (86.7)	0.536
Sometimes/never	12 (16)	9 (18.4)		8 (20)	4 (13.3)	
**Believes it is still possible to purchase flavored tobacco products even with SB 793**						
Always	36 (50)	26 (53.1)	0.853	20 (54.1)	6 (20)	**0.005**
Sometimes/never	36 (50)	23 (46.9)		17 (45.9)	24 (80)	
**Uses flavored tobacco products less with change in law in California**						
Always	35 (74.5)	20 (48.8)	**0.016**	29 (90.6)	20 (76.9)	0.274
Sometimes/never	12 (25.5)	21 (51.2)		3 (9.4)	6 (23.1)	
**Friends use flavored tobacco products less with change in law in California**						
Always	31 (59.6)	17 (34.7)	**0.017**	29 (80.5)	21 (77.8)	1
Sometimes/never	21 (39.6)	32 (65.3)		7 (19.4)	6 (22.2)	

SB 793: Senate Bill 793. ENDS: electronic nicotine delivery systems. SHA: secondhand aerosol. SHS: secondhand smoke.

* Statistical tests used were chi-squared.

Middle school students were similar to their mothers in terms of not allowing anyone to use tobacco products around them (95.0% vs 93.3%, p=1.0). Although we did not find any statistically significant differences between 7-day exposure to SHS and SHA between mothers and children for either high or middle school students, a high percentage of high school students (67%) who cited other exposures indicated that all these exposures happened at school (in the bathrooms or walkways). Conversely, mothers and middle school students indicated that their other exposures to SHS and SHA happened in the street or other outdoor venues, and none indicated that it was at school or work.

High school students were less likely to always avoid aerosol exposure compared to their mothers (74.0% vs 88.0%, p=0.056) (Table 2) although the results did not meet statistical significance. However, mothers of high school students were more likely to move away from SHS and/or SHA than their children (88.0% vs 70.0%, p=0.02) (Table 2). There were no statistically significant differences between middle school students and mothers in regard to SHS or SHA avoidance. Both reported a high rate of avoidance (92.5% and 96.7%, respectively, for SHA, and 92.5% and 93.3%, respectively, for avoidance of SHS and/or SHA (Table 2).

### Beliefs regarding use of tobacco products and risk of COVID-19

Mothers of high school students were more likely than high school students to believe that use of tobacco products is more dangerous in the context of COVID-19 (68.1% of mothers versus 48.0% of students, p=0.038). Similarly, mothers of high school students were also more likely to believe that smoking and using of ENDS product increase the risk of developing severe COVID-19 (57.8% of mothers, versus 34% of students, p=0.016). There was no difference between mothers of middle school students and middle school students on beliefs about the impact of tobacco product use on risk of COVID-19 and severe COVID-19. (Table 2)

### Legislation in California (SB 793) and behavior changes related to legislation

A high percentage of high and middle school students and their mothers agreed with SB 793, banning the sale of flavored tobacco products (84.0% of mothers of high school and 80.0% of mothers of middle school students; 81.6% of high school and 86.7% of middle school students) (Table 2). However, high school students did not think that SB 793 would change use patterns of flavored tobacco products for themselves or any friends compared with their mothers (51.2% of students believed the law would not be impactful, and 65.3% believed it would not be impactful for friends), compared with 25.5% of mothers who thought the impact would be minimal impactful for themselves or 39.6% for friends (p=0.016 and p=0.017, respectively) (Table 2).

## DISCUSSION

In this study of SHS and SHA exposures, we found approximately half of high school students and slightly less than half of middle school students had weekly exposures comparable with national level data from the 2015–2017 National Youth Tobacco Survey^5^. The on-going high level of exposure for students indicates an urgent need to focus on smoke and aerosol-free environments particularly in outdoor settings for Californians. Additionally, a high percentage of high school students reported SHS and/or SHA exposures compared with middle school students, particularly within school settings such as bathrooms, hallways, and outdoors areas. Similarly, a higher percentage of high school students indicated that they allowed others to use tobacco products around them at school than did middle school students or their parents. The high school students in our study were significantly older (17 years) versus the middle school students (11 years) and other studies have found a much higher percentage of tobacco product use among high versus middle school students^15^, as well as an accelerated increase of e-cigarette initiation from 11 to 17 years of age^16^.

### SB 793

Although SB 793 now prohibits the sale of flavored tobacco products in California, high school students surveyed often stated that the bill would make no difference for themselves or their peers in terms of having access to flavored tobacco products. High school students noted that they were able to obtain flavored vapes from other places, including from friends, online and smoke shops that continue to sell flavored tobacco products despite the new law. By contrast middle school students believed in the efficacy of the law to prevent flavored tobacco sales.

The ability for Bay Area high schoolers to easily access flavored tobacco products has been confirmed in studies from other parts of California. In a recent study more than half of flavored tobacco online transactions in San Diego were still delivered in spite of SB 793 and citywide ordinances preventing online sales of flavored tobacco^17^. San Francisco similarly has had an ordinance that bans online and mail order of flavored tobacco products since 2017, and recently sued Rogue Holdings, Swisher International and Northern Scandinavia for violating the ban and selling flavored Zyns (tobacco pouches) online^18^. California passed AB 3218 and SB 1230 in 2024 explicitly forbidding flavored tobacco online sales to ensure a further reduction in flavored tobacco use as online sales were not specifically mentioned in SB 793^19^. Meanwhile, we found two online sites that were advertising the sale of flavored tobacco products in SF^20,21^.

### COVID-19

High school students were less concerned about any interactions between SHS and SHA exposures and COVID-19 risk and severity of disease, compared with mothers and middle school students. Previous studies have found that SHS and SHA exposure is associated with more severe COVID-19 infection^22^, and it may be helpful in future educational campaigns to stress the risk associated with subsequent health issues including COVID-19 before students enter high school, prior to any initiation of tobacco use products^23^.

### Limitations

Limitations of this study include a small sample size and the inability to conduct any adjusted analyses. However, our population was relatively homogenous in terms of country of origin, participation in WIC and overall socioeconomic status^10,11^. Additionally, the focus of the study was on Spanish-speaking mothers and their US-born children in the San Francisco Bay Area, which may limit the generalizability of our findings to other populations including other Latino populations in the state of California. Lastly, as the study was cross-sectional, we were unable to determine causality between exposures, and self-reported data may have resulted in misclassification bias although the direction of the bias would have been non-differential.

## CONCLUSIONS

Additional studies are needed to assess SHA and SHS exposures in multi-racial and ethnic cohorts, as we assessed only Latino, US-born students and their predominantly foreign-born mothers. Future interventions should also focus on the early years of high school to prevent initiation, and work with school districts to prevent bathroom, hallway and other exposure areas during the school day. Other studies and interventions also need to ensure that online, and brick and mortar retailers comply with statewide and municipal legislation, and continue to monitor how and where students are accessing tobacco products.

## Data Availability

The data supporting this research are available from the authors on reasonable request.

## References

[1] Clodfelter R, Dutra LM, Bradfield B, et al. Results of the 2024 California Youth Tobacco Survey. Research Triangle Institute; 2025. Accessed May 29, 2026. https://cyts.rti.org/Content/CYTS-2024-Annual-Report.pdf

[2] Office of Environmental Health Hazard Assessment California Environmental Protection Agency. Analysis of Race/Ethnicity, Age, and CalEnviroScreen 3.0 Scores. Office of Environmental Health Hazard Assessment California Environmental Protection Agency; 2018. Accessed May 29, 2026. https://oehha.ca.gov/sites/default/files/media/downloads/calenviroscreen/document-calenviroscreen/raceageces3analysis.pdf

[3] California Legislative Information. Bill Text - SB-793 Flavored tobacco products. Accessed May 29, 2026. https://leginfo.legislature.ca.gov/faces/billTextClient.xhtml?bill_id=201920200SB793

[4] Jamal A, Park-Lee E, Birdsey J, et al. Tobacco product use among middle and high school students - National Youth Tobacco Survey, United States, 2024. MMWR Morb Mortal Wkly Rep. 2024;73(41):917-924. doi:10.15585/mmwr.mm7341a2 39418216 PMC11486349

[5] Gentzke AS, Wang TW, Cornelius M, et al. Tobacco product use and associated factors among middle and high school students - National Youth Tobacco Survey, United States, 2021. MMWR Surveill Summ. 2022;71(5):1-29. doi:10.15585/mmwr.ss7105a1 PMC8923300 35271557

[6] Chaffee BW, Donaldson CD, Couch ET, et al. Flavored tobacco product use among california adolescents before and immediately after a statewide flavor ban. Nicotine Tob Res. 2025;27(6):1035-1042. doi:10.1093/ntr/ntae261 39529400 PMC12095798

[7] Donaldson SI, Beard TA, Colonna R, et al. Online purchase attempts of flavored e-cigarettes to minors in california before and after senate bill 793. JAMA Netw Open. 2023;6(12):e2348749. doi:10.1001/jamanetworkopen.2023.48749 38127352 PMC10739096

[8] Denlinger-Apte R, Suerken CK, Ross JC, et al. Decreases in smoking and vaping during COVID-19 stay-at-home orders among a cohort of young adults in the United States. Prev Med. 2022;156:106992. doi:10.1016/j.ypmed.2022.106992 35149114 PMC8824729

[9] Kreslake JM, O'Connor KM, Liu M, Vallone DM, Hair E. A resurgence of e-cigarette use among adolescents and young adults late in the COVID-19 pandemic. PLoS One. 2023;18(3):e0282894. doi:10.1371/journal.pone.0282894 36989261 PMC10057832

[10] Wojcicki JM, Holbrook K, Lustig RH, et al. Chronic maternal depression is associated with reduced weight gain in latino infants from birth to 2 years of age. PLoS One. 2011;6(2):e16737. doi:10.1371/journal.pone.0016737 21373638 PMC3044151

[11] Ville AP, Heyman MB, Medrano R, Wojcicki JM. Early antibiotic exposure and risk of childhood obesity in latinos. Child Obes. 2017;13(3):231-235. doi:10.1089/chi.2016.0235 28165758 PMC5444413

[12] Escobar M, Mendez AD, Encinas MR, Villagomez S, Wojcicki JM. High food insecurity in Latinx families and associated COVID-19 infection in the Greater Bay Area, California. BMC Nutr. 2021;7(1):23. doi:10.1186/s40795-021-00419-1 34112257 PMC8192129

[13] Prochaska JJ, Grossman W, Young-Wolff KC, Benowitz NL. Validity of self-reported adult secondhand smoke exposure. Tob Control. 2015;24(1):48-53. doi:10.1136/tobaccocontrol-2013-051174 23997071 PMC4458852

[14] Lewis-Esquerre JM, Rodrigue JR, Kahler CW. Development and validation of an adolescent smoking consequences questionnaire. Nicotine Tob Res. 2005;7(1):81-90. doi:10.1080/14622200412331328475 15804680

[15] Birdsey J, Cornelius M, Jamal A, et al. Tobacco product use among u.s. middle and high school students - National Youth Tobacco Survey, 2023. MMWR Morb Mortal Wkly Rep. 2023;72(44):1173-1182. doi:10.15585/mmwr.mm7244a1 37917558 PMC10629751

[16] Chen X, Yu B, Wang Y. Initiation of electronic cigarette use by age among youth in the U.S. Am J Prev Med. 2017;53(3):396-399. doi:10.1016/j.amepre.2017.02.011 28372920

[17] Harati RM, Ellis SE, Satybaldiyeva N, et al. Online retailer nonadherence to age verification, shipping, and flavor restrictions on e-cigarettes. JAMA. 2024;332(24):2113-2114. doi:10.1001/jama.2024.21597 39527068 PMC11555574

[18] Greschler G. Zyn sellers to pay city $3 million for violating bad on flavored nicotine products. The San Francisco Standard; 2025. Accessed May 29, 2026. https://sfstandard.com/2025/10/24/san-francisco-zyn-settlement-flavored-tobacco-ban/

[19] Public Health Law Center. 9/30/24-AB 3218 and SB 1230: The Next Step in California’s Flavor Law. Accessed May 29, 2026. https://www.publichealthlawcenter.org/commentary/240930/9/30/24-ab-3218-and-sb-1230-next-step-californias-flavor-law

[20] Saucey. Nicotine & Tobacco Delivery Near You - Online Delivery Near You. Accessed May 29, 2026. https://saucey.com/nicotine-tobacco

[21] The Dab Lab. The DAB lab: The best online headshop. Accessed May 29, 2026. https://www.thedablab.com/

[22] Kishore S, Shah V, Bera OP, et al. Risk of secondhand smoke exposure and severity of COVID-19 infection: Multicenter case-control study. Front Public Health. 2023;11:1210102. doi:10.3389/fpubh.2023.1210102 37601179 PMC10435989

[23] Aldukhail S, Alabdulkarim A, Agaku IT. Population-level impact of 'The Real Cost' campaign on youth smoking risk perceptions and curiosity, United Sates 2018-2020. Tob Induc Dis. 2023;21:162. doi:10.18332/tid/174900 38090739 PMC10714412

